# Coconut Oil Aggravates Pressure Overload-Induced Cardiomyopathy without Inducing Obesity, Systemic Insulin Resistance, or Cardiac Steatosis

**DOI:** 10.3390/ijms18071565

**Published:** 2017-07-18

**Authors:** Ilayaraja Muthuramu, Ruhul Amin, Andrey Postnov, Mudit Mishra, Frank Jacobs, Olivier Gheysens, Paul P. Van Veldhoven, Bart De Geest

**Affiliations:** 1 Centre for Molecular and Vascular Biology, Department of Cardiovascular Sciences, Catholic University of Leuven, Leuven 3000, Belgium; ilayaraja.muthuramu@kuleuven.be (I.M.); rbio5226@gmail.com (R.A.); mudit.mishra@kuleuven.be (M.M.); FJACOBS1@its.jnj.com (F.J.); 2 Nuclear Medicine & Molecular Imaging, Department of Imaging & Pathology, Catholic University of Leuven, Leuven 3000, Belgium; postnov.email@gmail.com (A.P.); olivier.gheysens@uzleuven.be (O.G.); 3 Laboratory of Lipid Biochemistry and Protein Interactions, Department of Cellular and Molecular Medicine, Catholic University of Leuven, Leuven 3000, Belgium; Paul.VanVeldhoven@kuleuven.be

**Keywords:** cardiac hypertrophy, coconut oil, nutrition, heart failure, pressure overload, cardiac function, oxidative stress, saturated fatty acids, metabolism

## Abstract

Studies evaluating the effects of high-saturated fat diets on cardiac function are most often confounded by diet-induced obesity and by systemic insulin resistance. We evaluated whether coconut oil, containing C12:0 and C14:0 as main fatty acids, aggravates pressure overload-induced cardiomyopathy induced by transverse aortic constriction (TAC) in C57BL/6 mice. Mortality rate after TAC was higher (*p* < 0.05) in 0.2% cholesterol 10% coconut oil diet-fed mice than in standard chow-fed mice (hazard ratio 2.32, 95% confidence interval 1.16 to 4.64) during eight weeks of follow-up. The effects of coconut oil on cardiac remodeling occurred in the absence of weight gain and of systemic insulin resistance. Wet lung weight was 1.76-fold (*p* < 0.01) higher in coconut oil mice than in standard chow mice. Myocardial capillary density (*p* < 0.001) was decreased, interstitial fibrosis was 1.88-fold (*p* < 0.001) higher, and systolic and diastolic function was worse in coconut oil mice than in standard chow mice. Myocardial glucose uptake was 1.86-fold (*p* < 0.001) higher in coconut oil mice and was accompanied by higher myocardial pyruvate dehydrogenase levels and higher acetyl-CoA carboxylase levels. The coconut oil diet increased oxidative stress. Myocardial triglycerides and free fatty acids were lower (*p* < 0.05) in coconut oil mice. In conclusion, coconut oil aggravates pressure overload-induced cardiomyopathy.

## 1. Introduction

Higher plasma levels of saturated fatty acids (SFA), especially palmitic acid (C16:0) and myristic acid (C14:0), were independently associated with incident heart failure in both men and women in the Atherosclerosis Risk in Communities Study [1]. Experimental animal studies suggest that dietary SFA may directly promote the development of heart failure by inducing lipotoxicity [2]. However, most studies on the role of SFA in cardiac dysfunction have been based on obesogenic high-fat diets. Obesity-associated inflammation and systemic insulin resistance confound experimental investigations designed to study the causal role of SFA in cardiac dysfunction and heart failure. Indeed, systemic insulin resistance and associated chronic hyperinsulinemia may play a prominent direct role in the development of cardiac dysfunction. Whereas the insulin/phosphatidylinositol-4,5-bisphosphate 3-kinase (PI3K)/Akt axis is involved in normal cardiac growth and physiological hypertrophy [3,4], chronic hyperinsulinemia stimulates angiotensin II signaling that is involved in pathological hypertrophy [5]. Mismatch between cardiomyocyte size and vascularity may contribute to the transition from cardiac hypertrophy to heart failure [6].

The effect of dietary SFA on the development of heart failure may be highly dependent on SFA chain length. The myristate-containing ceramide species C_14_-ceramide has been implicated in the pathogenesis of lipotoxic cardiomyopathy in a milk-fat based diet model that was associated with an approximately 20% increase in body weight [2,7]. A cocoa butter derived high-fat diet (containing mainly palmitate (C16:0) and stearate (C18:0)) resulted in minor [8] or no [9] effects on body weight and did not have major effects on cardiac structure and function compared to a low-fat diet in sham-operated mice and in mice with pressure overload induced by transverse aortic constriction [8,9].

The objective of the current study was to evaluate whether coconut oil, which contains lauric acid (C12:0) and myristic acid (C14:0) as main fatty acids, modifies the development of pressure overload-induced cardiomyopathy induced by transverse aortic constriction (TAC) in C57BL/6 mice without inducing weight gain or systemic insulin resistance.

## 2. Results

### 2.1. The 0.2% Cholesterol 10% Coconut Oil (CC) Diet Significantly Increases the Mortality Rate after TAC

**Figure 1:**
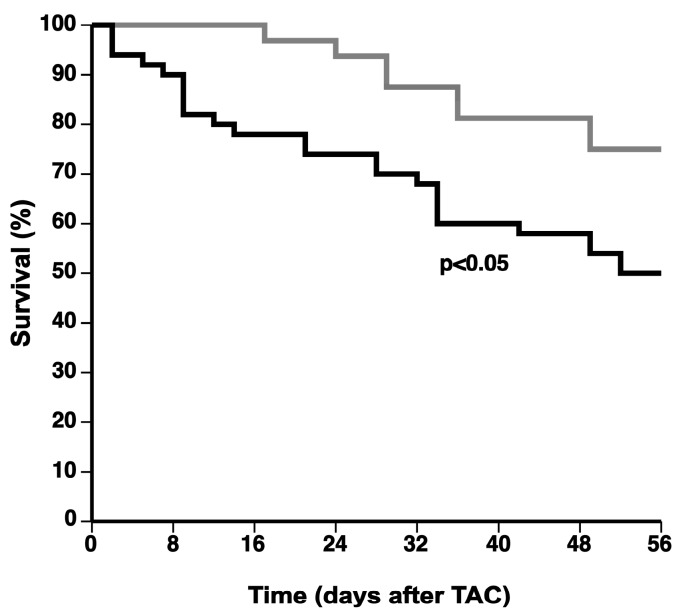
Kaplan–Meier survival curves during an eight weeks follow-up period after TAC. Female C57BL/6 mice on SC diet (grey line) or CC diet (black line) are compared. The Day 0 time-point corresponds to TAC intervention at the age of 17 weeks. Survival analysis was performed by log-rank test.

The CC diet was initiated at the age of 12 weeks in female C57BL/6 mice and was sustained in CC diet mice for the entire duration of the experiment. The CC diet did not alter lipoprotein cholesterol levels compared to the standard chow (SC) diet during the entire duration of the experiment (Table 1). TAC was performed at the age of 17 weeks to induce pressure overload. Comparison of Kaplan–Meier survival curves showed a significantly (*p* < 0.05) higher mortality rate in CC diet TAC mice compared to SC diet TAC mice (hazard ratio for mortality 2.32, 95% CI 1.16 to 4.64) during eight weeks of follow-up (Figure 1). Sham operation did not result in any mortality (data not shown).

### 2.2. Increased Lung Congestion and More Pronounced Right Ventricular Hypertrophy after TAC in CC Diet Mice

No significant difference in heart, lung, or liver weight was observed between the different sham groups (Table 2). The heart weight was 2.01-fold (*p* < 0.001) higher in SC diet TAC mice and 2.47-fold (*p* < 0.001) higher in CC diet TAC mice compared to respective sham groups. There was a 1.21-fold (*p* < 0.001) increase in the heart weight in CC diet TAC mice compared to SC diet TAC mice. Left ventricular weight and right ventricular weight were 1.10-fold (*p* = NS) and 1.44-fold (*p* < 0.001) higher, respectively, in CC diet TAC mice than in SC diet TAC mice. The lung weight was 1.76-fold (*p* < 0.01) increased in CC diet TAC mice compared to SC diet TAC mice. Taken together, these data suggest more pronounced left ventricular failure in CC diet TAC mice as evidenced by increased lung congestion and right ventricular hypertrophy.

### 2.3. The CC Diet Results in Lower Capillary Density and Relative Vascularity and Increased Interstitial and Perivascular Fibrosis after TAC

Anterior wall thickness and septal wall thickness were similarly increased in both TAC groups compared to respective sham groups (Table 3). Cardiomyocyte cross-sectional area in the left ventricle was 1.11-fold (*p* = NS) higher in the CC diet TAC group compared to the SC diet TAC group. The cardiomyocyte cross-sectional area in the right ventricle was similar in SC diet sham mice (160 ± 2 µm^2^; *n* = 10) and in CC diet sham mice (166 ± 5 μm^2^; *n* = 10) and was increased by 1.36-fold (*p* < 0.001) and by 1.89-fold (*p* < 0.001) in SC diet TAC mice (218 ± 12 μm^2^; *n* = 10) and CC diet TAC mice (313 ± 12 µm^2^; *n* = 10), respectively, compared to respective sham groups. The right ventricular cardiomyocyte cross-sectional area was 1.44-fold (*p* < 0.001) higher in CC diet TAC mice than in SC diet TAC mice. These measurements parallel right ventricular weight measurements and are consistent with more pronounced right ventricular hypertrophy in CC diet TAC mice compared to SC diet TAC mice.

**Figure 2:**
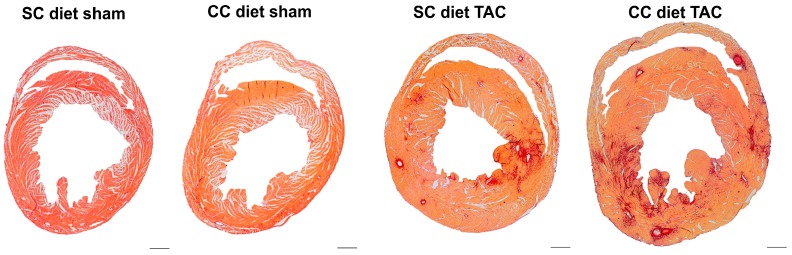
Representative Sirius red-stained cross-sections of sham hearts and TAC hearts at Day 56 after intervention. Scale bar represents 1 mm.

Representative Sirius red-stained cross-sections of sham hearts and TAC hearts at Day 56 after operation are shown in Figure 2.

**Figure 3:**
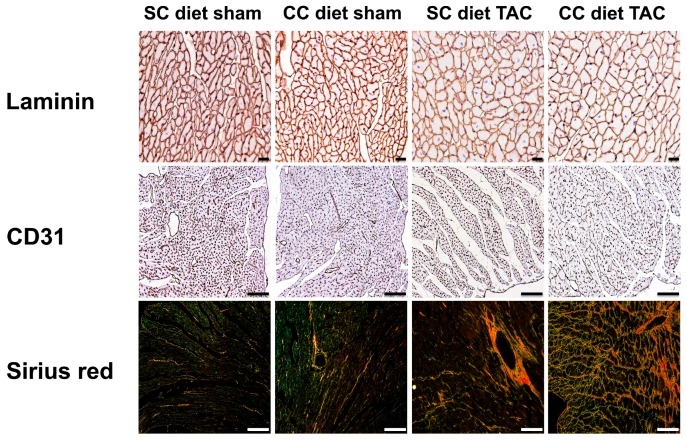
(Immuno) histochemical analysis of the myocardium of sham mice and TAC mice at Day 56 after operation. Representative photomicrographs show laminin-stained cardiomyocytes, CD31-positive capillaries, and Sirius red-stained interstitial collagen viewed under polarized light. Scale bar represents 50 μm.

Reduced vessel density and interstitial fibrosis are markers of pathological hypertrophy. Capillary density (*p* < 0.001) and relative vascularity (*p* < 0.001) were significantly decreased and interstitial fibrosis was 1.88-fold (*p* < 0.001) higher in CC diet TAC mice than in SC diet TAC mice. Strikingly, interstitial fibrosis was 3.37-fold (*p* < 0.05) higher in CC diet sham mice compared to SC diet sham mice (Table 3). Perivascular fibrosis was 1.40-fold (*p* < 0.001) higher in CC diet TAC mice than in SC diet TAC mice and was also more pronounced (*p* < 0.05) in CC diet sham than in SC diet sham mice. Figure 3 shows representative photomicrographs of laminin-stained cardiomyocytes, CD31-positive capillaries, and Sirius red-stained interstitial collagen viewed under polarized light.

Apoptotic cells, identified as cleaved caspase-3-positive cells, were barely detectable in the myocardium of sham mice (Table 3). Compared to SC diet TAC mice, the number of cleaved caspase-3-positive cells was 1.62-fold (*p* < 0.001) higher in CC diet TAC mice.

### 2.4. Differential Activation of Pro-Hypertrophic Signaling Pathways in SC Diet Mice and CC Diet Mice after TAC

**Figure 4:**
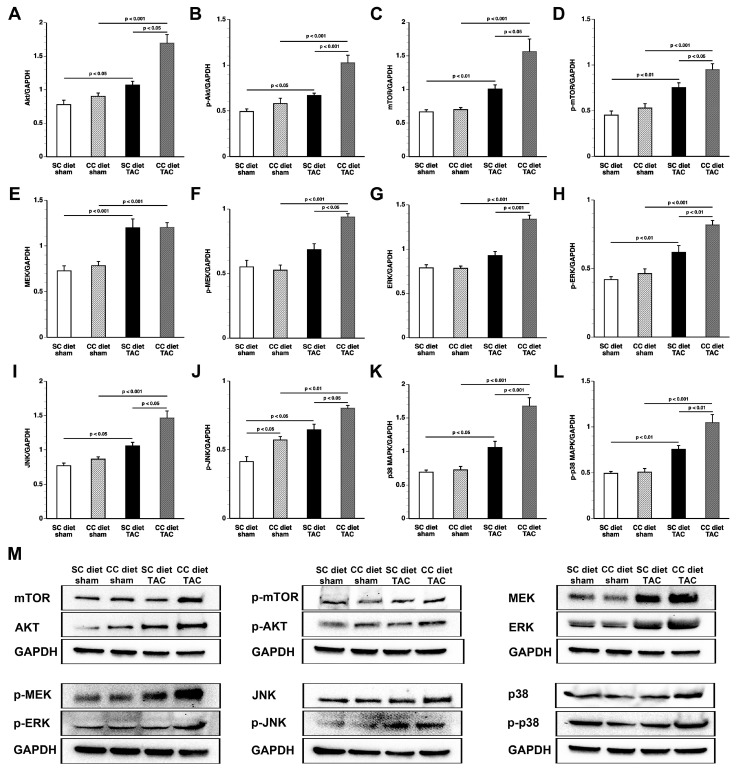
Quantification of pro-hypertrophic myocardial proteins by Western blot. Intensities of immunoreactive bands on Western blots were quantified by densitometric analysis. Bar graphs illustrating: Akt (**A**); p-Akt (**B**); mTOR (**C**); p-mTOR (**D**); MEK (**E**); p-MEK (**F**); ERK (**G**); p-ERK (**H**); JNK (**I**); p-JNK (**J**); p38 (**K**); and p-p38 (**L**) protein levels quantified by Western blot in the myocardium of SC diet sham (*n* = 10), CC diet sham (*n* = 10), SC diet TAC (*n* = 10), and CC diet TAC (*n* = 10) mice eight weeks after operation. All protein levels were normalized to the glyceraldehyde-3-phosphate dehydrogenase (GAPDH) protein level. Representative images of Western blots are shown in panel (**M**).

Activation of several pro-hypertrophic signaling pathways was more pronounced in CC diet TAC mice than in SC diet TAC mice (Figure 4). Myocardial Akt and p-Akt levels were 1.58-fold (*p* < 0.05) higher and 1.54-fold (*p* < 0.001) higher, respectively, in CC diet TAC mice than in SC diet TAC mice. Similarly, myocardial levels of mammalian or mechanistic target of rapamycin (mTOR), involved in cell growth, autophagy, and metabolism, and of p-mTOR were significantly (*p* < 0.05) increased in CC diet TAC mice compared to SC diet TAC mice. Myocardial protein levels of p-mitogen-activated protein kinase (MAPK) kinase (p-MEK) protein levels, of extracellular signal-regulated kinase (ERK), p-ERK, c-Jun N-terminal kinase (JNK), p-JNK, p38 MAPK (p38), and p-p38 MAPK levels were all significantly higher in CC diet TAC mice than in SC diet TAC mice. In addition, myocardial levels of p-JNK were 1.38-fold (*p* < 0.05) higher in CC diet sham mice compared to SC diet sham mice. Representative images of Western blots are shown in Figure 4M. Taken together, differential activation of pro-hypertrophic signaling pathways in SC diet mice and CC diet mice after TAC may have contributed to more prominent pathological remodeling in the latter.

### 2.5. The CC Diet Results in Increased Myocardial TGF-*β*1 and Pro-Fibrotic Signaling Molecules in the Presence and Absence of Pressure Overload

**Figure 5:**
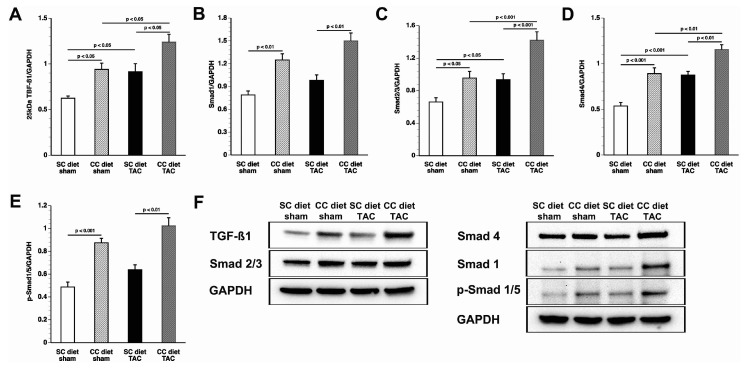
Quantification of TGF-β1 and pro-fibrotic signaling molecules in the myocardium by Western blot. Intensities of immunoreactive bands on Western blots were quantified by densitometric analysis. Bar graphs illustrating the 25-kD isoform of: TGF-β1 (**A**); Smad1 (**B**); Smad2/3 (**C**); Smad 4 (**D**); and p-Smad1/5 (**E**) protein levels quantified by Western blot in the myocardium of SC diet sham (*n* = 10), CC diet sham (*n* = 10), SC diet TAC (*n* = 10), and CC diet TAC (*n* = 10) mice eight weeks after intervention. All protein levels were normalized to the glyceraldehyde-3-phosphate dehydrogenase (GAPDH) protein level. Representative images of Western blots are shown in panel (**F**).

As shown in Table 3, the CC diet potentiated fibrosis in both sham mice and TAC mice. Myocardial levels of the 25-kD isoform of transforming growth factor (TGF)-β1 were 1.50-fold (*p* < 0.05) higher and 1.36-fold (*p* < 0.05) higher in CC diet sham and CC diet TAC mice, respectively, compared to respective SC diet groups (Figure 5). Similar differences were observed for the 12.5 kD isoform of TGF-β1 (data not shown). Smad1 levels in the myocardium were 1.58-fold (*p* < 0.05) higher and 1.53-fold (*p* < 0.05) higher in CC diet sham and CC diet TAC mice, respectively, compared to respective SC diet groups. Similar increases were observed for Smad2/3, Smad4, and p-Smad1/5 (Figure 5). Representative images of Western blots are shown in Figure 5F.

### 2.6. The CC Diet Significantly Worsens Cardiac Function in the Presence of Pressure Overload

**Figure 6:**
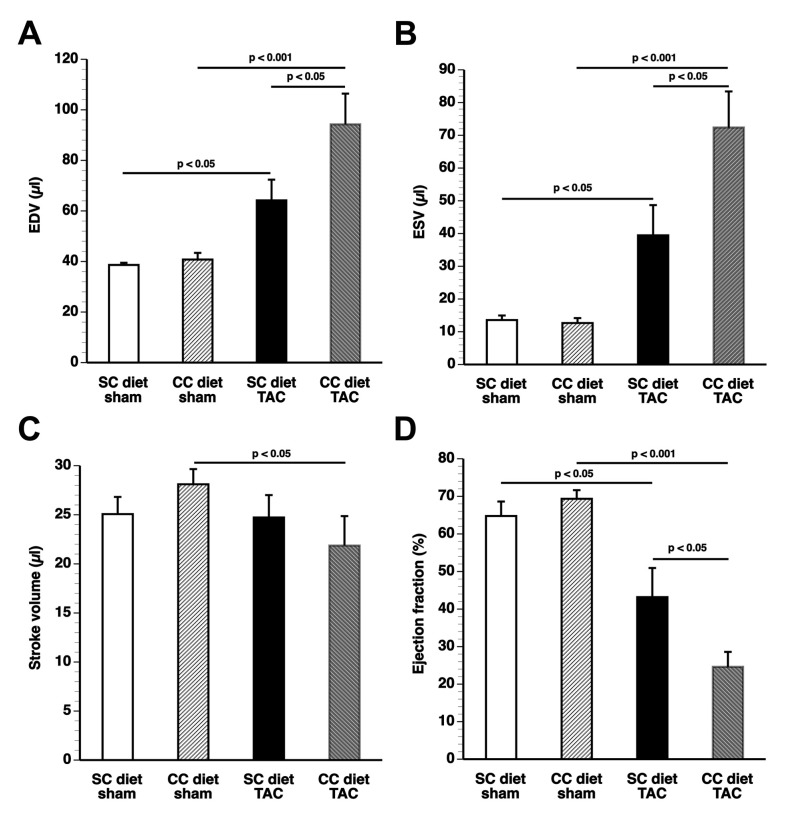
Quantification of (**A**) end-diastolic volume (EDV), (**B**) end-systolic volume (ESV), (**C**) stroke volume, and (**D**) ejection fraction in sham mice and TAC mice by electrocardiography (ECG)-gated micro-PET. The CC diet was initiated at the age of 12 weeks. Sham operation or TAC was performed at the age of 17 weeks. ECG-gated PET imaging was performed eight weeks later. Data represent means ± SEM (*n* = 16).

The peak rate of isovolumetric contraction (dP/dt max) and the absolute value of the peak rate of isovolumetric relaxation (dP/dt min) were significantly (*p* < 0.05) lower in CC diet TAC mice compared to SC diet TAC mice (Table 4). Furthermore, the time constant of isovolumetric relaxation was significantly (*p* < 0.05) higher in CC diet sham mice than in SC diet sham mice, indicating impaired isovolumetric relaxation. The quantification of end-diastolic volume (EDV), end-systolic volume (ESV), stroke volume, and ejection fraction by electrocardiography (ECG)-gated Positron Emission Tomography (PET) is shown in Figure 6. The EDV was increased by 1.66-fold (*p* < 0.05) in SC diet TAC mice and by 2.31-fold (*p* < 005) in CC diet TAC mice compared to respective sham groups. The EDV was 46.9% (*p* < 0.05) higher in CC diet TAC mice than in SC diet TAC mice. The ESV was markedly increased in TAC groups compared to respective sham groups and was 1.83-fold (*p* < 0.05) higher in CC diet TAC mice than in SC diet TAC mice. The stroke volume was reduced by 22.2% (*p* < 0.05) in CC diet TAC mice compared to CC diet sham mice. The ejection fraction was significantly (*p* < 0.05) lower in CC diet TAC mice than in SC diet TAC mice. Taken together, the deterioration of cardiac function after TAC is significantly more prominent in CC diet mice than in SC diet mice.

### 2.7. The CC Diet Aggravates Metabolic Remodeling Induced by Pressure Overload

**Figure 7:**
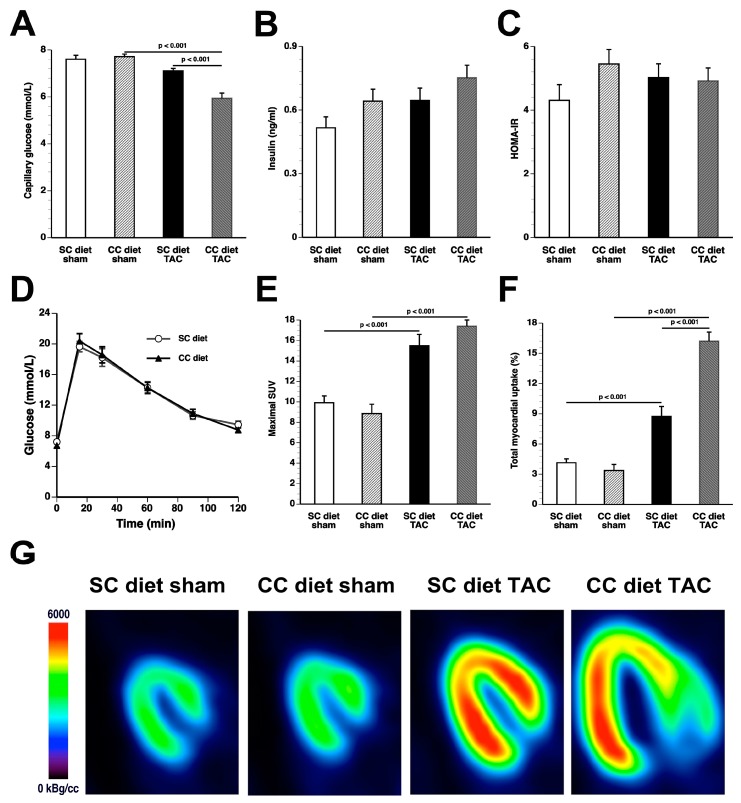
The CC diet induces more pronounced metabolic remodeling after TAC. Capillary glucose (**A**); plasma insulin levels (**B**); and HOMA-IR values (**C**) in SC diet sham, CC diet sham, SC diet TAC, and CC diet TAC mice eight weeks after intervention. Blood glucose levels following an intraperitoneal glucose tolerance test in SC diet mice and in CC diet mice (**D**). Quantification of glucose uptake in the myocardium determined by micro-PET as shown by the: maximal standardized uptake (SUV) value (**E**); and total myocardial uptake (% of injected dose) (**F**) eight weeks after sham operation or after TAC. All data represent means ± SEM (*n* = 16). Representative micro-PET images illustrating the uptake of (^18^F)-fluorodeoxyglucose (FDG) in the myocardium of sham mice and TAC mice at Day 56 after operation are shown in panel (**G**).

Whole blood glucose in CC diet TAC mice was 23.4% (*p* < 0.001) and 16.3% (*p* < 0.001) lower than in CC diet sham mice and SC diet TAC mice, respectively (Figure 7A). Differences in plasma insulin levels (Figure 7B) and in homeostatic model assessment-insulin resistance (HOMA-IR) values (Figure 7C) did not reach statistical significance, suggesting absence of systemic insulin resistance in CC diet mice. Blood glucose levels following an intraperitoneal glucose tolerance test were similar at 0, 15, 30, 60, 90, and 120 min in SC diet mice (*n* = 17) and in CC diet mice (*n* = 17), indicating absence of glucose intolerance in CC diet mice (Figure 7D).

TAC was associated with a significantly increased glucose uptake in the myocardium as quantified by micro-PET imaging using (^18^F)-fluorodeoxyglucose (FDG) as a tracer. The maximal standardized uptake value (SUV) was 1.56-fold (*p* < 0.001) higher in SC diet TAC mice and 1.97-fold (*p* < 0.001) higher in CC diet TAC mice compared to respective sham groups (Figure 7E). The total accumulation of glucose in the myocardium was 2.11-fold (*p* < 0.001) higher in SC diet TAC mice and 4.80-fold (*p* < 0.001) higher in CC diet TAC mice compared to respective sham groups (Figure 7F). In addition, accumulation of glucose in the myocardium determined by micro-PET was 1.86-fold (*p* < 0.001) higher in CC diet TAC mice compared to SC diet TAC mice. Additional parameters of glucose uptake in the myocardium and in the left quadriceps are shown in Table 5. Representative micro-PET images illustrating the uptake of (^18^F)-FDG in the myocardium of SC diet mice and CC diet mice are shown in Figure 7G.

**Figure 8:**
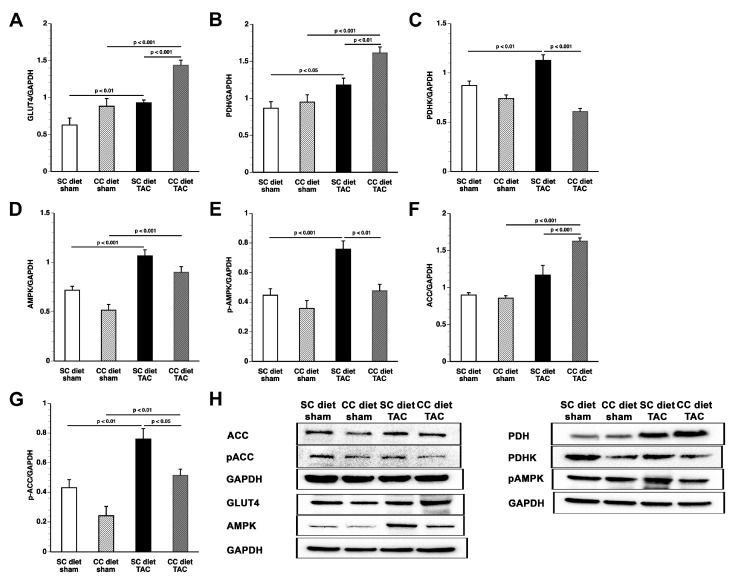
Quantification of metabolic proteins by Western blot. Intensities of immunoreactive bands on Western blots were quantified by densitometric analysis. Bar graphs illustrating: GLUT4 (**A**); PDH (**B**); PDHK (**C**); AMPK (**D**); p-AMPK (**E**); acetyl-CoA carboxylase (AAC) (**F**); and p-ACC (**G**) protein levels quantified by Western blot in the myocardium of SC diet sham (*n* = 10), CC diet sham (*n*=10), SC diet TAC (*n* = 10), and CC diet TAC (*n* = 10) mice eight weeks after operation. All protein levels were normalized to the glyceraldehyde-3-phosphate dehydrogenase (GAPDH) protein level. Representative images of Western blots are shown in panel (**H**).

Pressure overload significantly increased myocardial GLUT4 protein levels and pyruvate dehydrogenase (PDH) levels (Figure 8). Myocardial GLUT4 and PDH levels were 1.55-fold (*p* < 0.001) and 1.37-fold (*p* < 0.01) higher in CC diet TAC mice than in SC diet TAC mice. Myocardial protein levels of PDH kinase (PDHK), which inactivates PDH, were reduced by 46.0% (*p* < 0.001) in control TAC mice compared to control sham mice and were 2.24-fold (*p* < 0.001) higher in CC diet TAC mice compared to SC diet TAC mice. Taken together, these data suggest that increased glucose uptake is accompanied by increased glucose oxidation in CC diet TAC mice.

Myocardial AMP-activated protein kinase (AMPK) protein levels were significantly (*p* < 0.001) increased in SC diet TAC mice and CC diet TAC mice compared to respective sham groups. Levels of p-AMPK were 37.0% (*p* < 0.01) lower in CC diet TAC mice than in SC diet TAC mice. Myocardial acetyl-CoA carboxylase (ACC) levels were 1.39-fold (*p* < 0.001) higher in the CC diet TAC mice than in the SC diet TAC mice. Consistent with lower p-AMPK levels in CC diet TAC mice, the phosphorylated, inactive form of ACC was reduced by 32.4% (*p* < 0.05) compared to SC diet TAC mice. Taken together, these data suggest more pronounced inhibition of fatty acid oxidation in CC diet TAC mice than in SC diet TAC mice. Representative images of Western blots are shown in Figure 8H.

### 2.8. The CC Diet Increases Oxidative Stress

**Figure 9:**
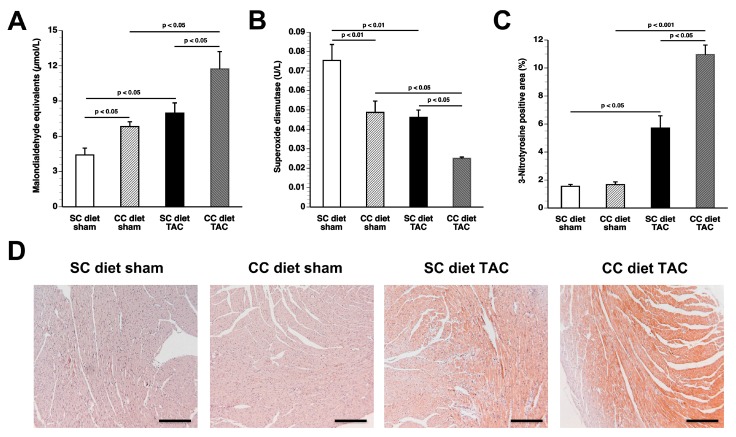
Quantification of oxidative stress in sham mice and in TAC mice at Day 56 after operation: sham mice and TAC mice are indicated by open bars and closed bars, respectively: (**A**) Plasma TBARS expressed as plasma malondialdehyde equivalents; (**B**) superoxide dismutase activity; (**C**) percentage of 3-nitrotyrosine-positive myocardial area; and (**D**) representative photomicrographs showing myocardial sections stained for 3-nitrotyrosine. All data represent means ± SEM (*n* = 16). Scale bar represents 100 µm.

Plasma thiobarbituric acid reactive substances (TBARS) were 1.80-fold (*p* < 0.05) higher in SC diet TAC mice and 1.72-fold (*p* < 0.05) higher in CC diet TAC mice compared to respective sham groups (Figure 9A). The CC diet resulted in significantly (*p* < 0.05) increased plasma TBARS in both the presence and absence of pressure overload (Figure 9A). Plasma superoxide dismutase activity was significantly reduced in TAC mice compared to respective sham groups and in CC diet mice compared to respective SC diet mice (Figure 9B). Compared to respective sham groups, the 3-nitrotyrosine-positive area (%) was increased by 3.68-fold (*p* < 0.05) and by 6.55-fold (*p* < 0.001) in SC diet TAC mice and in CC diet TAC mice, respectively (Figure 9C). The 3-nitrotyrosine-positive area was 1.92-fold (*p* < 0.05) higher in CC diet TAC mice than in SC diet TAC mice, indicating increased nitro-oxidative stress. Representative 3-nitrotyrosine-stained myocardial sections are shown in Figure 9D.

### 2.9. The CC Diet Does Not Result in Cardiac Steatosis

Myocardial lipid levels eight weeks after sham operation or after TAC in C57BL/6 mice fed the SC diet or the CC diet are shown in Table 6. No significant difference in the content of major myocardial lipid classes was observed between both sham groups. The sphingomyelin/phospholipid molar ratio was 1.37-fold (*p* < 0.01) increased in CC diet TAC mice compared to SC diet TAC mice. Myocardial triglyceride content and free fatty acid content were 41.8% (*p* < 0.05) and 48.0% (*p* < 0.05) lower, respectively, in CC diet TAC mice than in SC diet TAC mice (Table 6).

## 3. Discussion

The main findings of the current study are that: (1) features of pathological hypertrophy following pressure overload in mice fed coconut oil are more prominent compared to SC diet mice as evidenced by striking myocardial fibrosis, a sharper decline in capillary density, and increased apoptosis in the myocardium; (2) the deterioration of cardiac function after TAC is significantly more pronounced in CC diet mice than in SC diet mice and results in more marked heart failure as evidenced by the higher lung weight and the prominent right ventricular hypertrophy; (3) pressure overload induces more marked metabolic remodeling in CC diet mice as evidenced by higher glucose uptake, higher PDH levels, and higher ACC levels; and (4) the effects of dietary coconut oil on structural, functional, and metabolic remodeling occur in the absence of weight gain, of systemic insulin resistance, and of cardiac steatosis.

Coconut oil is an edible oil that is present in the meat of the coconut and extracted from the fruit of the coconut tree (*Cocos nucifera*). To exclude the possibility that the addition of 0.2% cholesterol to the diet modifies the effects of coconut oil, the effect of a 10% coconut oil diet without cholesterol on cardiac structure and function was evaluated. We did not observe any significant difference between 10% coconut oil-fed mice and CC diet mice (data not shown). Coconut oil predominantly contains medium-chain SFA: caproic acid (C6:0) 1%, caprylic acid (C8:0) 9%, capric acid (C10:0) 7%, and lauric acid (C12:0) 47% of the overall fatty acid composition [10]. Medium-chain fatty acids do not rely on membrane transporters for their uptake into cells and mitochondria [11,12]. They are directly activated in the mitochondrial matrix by medium-chain acyl-CoA synthetase prior to β-oxidation. The currently available evidence suggests potential beneficial effects of medium-chain fatty acids on the myocardium [11,13,14,15,16]. Therefore, it is rather unlikely that medium-chain fatty acids play a role in the coconut oil-induced pathological hypertrophy and remodeling. The large majority of lauric acid is transported directly to the liver via the portal vein whereas a minor part is reformed into new triglycerides and incorporated into chylomicrons, which enter the lymphatic system and reach the blood via the *ductus thoracicus* [17]. Lauric acid is rapidly oxidized in the liver and may induce the production of ketone bodies. Whereas ketone bodies may be used as fuel in advanced heart failure [18,19,20], there is no evidence for a detrimental role of ketone bodies on the myocardium [20].

Besides medium-chain fatty acids and the unsaturated fatty acids oleic acid (C18:1) (6%) and linoleic acid (C16:2, n-6) (1%), coconut oil further contains the long-chain SFA myristic acid (C14:0) (17%), palmitic acid (C16:0) (8%), and stearic acid (C18:0) (3%). The myristate-containing ceramide species C_14_-ceramide has been implicated in the pathogenesis of lipotoxic cardiomyopathy [2]. The potential pivotal role of myristic acid (C14:0) and palmitic acid (C16:0) is in line with epidemiological data [1].

Whereas coconut oil did not alter myocardial triacylglycerols in sham mice, a significant decline of triacylglycerols was observed in CC diet TAC mice compared to SC diet TAC mice. Cardiac steatosis typically occurs in the presence of obesity, insulin resistance, and diabetes mellitus. The CC diet did not induce obesity, diabetes mellitus, or impaired glucose tolerance. Furthermore, based on HOMA-IR values, systemic insulin resistance was absent. Although the hyperinsulinemic-euglycemic clamp is the gold-standard method to assess insulin sensitivity, the application of this technique in mice is challenging because of issues such as high-cost, need for pump-infusion equipment, and considerable expertise [21]. HOMA-IR values as surrogate measure provide a reasonable and reliable approximation of formal measures of insulin resistance when applied to rats and mice as they do in humans [22,23,24].

The absence of cardiac steatosis does not exclude the presence of lipotoxic molecules in the heart. Triacylglycerols, the main lipid stored in lipid droplets, are themselves not thought to be harmful [25]. Specific phospholipid and sphingolipid species, rather than diacylglycerols and triacylglycerols, may modulate the structural, metabolic, and functional effects of coconut oil. A significant decrease in phospholipids and a significant increase in sphingomyelin were present in CC diet TAC mice compared to SC diet TAC mice. Although these alterations may be the consequence of more prominent pathological remodeling and of more pronounced deterioration of cardiac function, specific lipid mediators are expected to be on the causal pathway between the CC diet and cardiac disease. Strikingly, the CC diet induced increased perivascular and interstitial fibrosis in sham mice and also resulted in deterioration of the isovolumetric relaxation in sham mice, indicating pressure overload-independent effects of the CC diet.

According to an often-proposed paradigm, the pressure-overloaded heart reverts toward a fetal-like metabolic profile, characterized by a decrease in fatty acid oxidation concomitant with an increased reliance on carbohydrates for oxidative energy metabolism [26]. Glucose uptake was nearly doubled in CC diet TAC mice compared to SC diet TAC mice notwithstanding a similar degree of left ventricular hypertrophy. The mTOR pathway plays a key role in sensing and integrating multiple environmental signals [27]. The mTOR complex 1 (mTORC1) promotes protein synthesis and cell growth, inhibits autophagy, and results in increased glucose oxidation and reduced fatty acid oxidation [28,29]. Increased mTOR and p-mTOR in CC diet TAC mice compared to SC diet TAC mice may be a critical mediator of many of the observed structural and metabolic effects.

In conclusion, the current study shows that coconut oil exerts profound effects on murine cardiac structure and function both in the presence and absence of pressure overload. Coconut oil induces oxidative stress and myocardial fibrosis even in the absence of pressure overload. Whether coconut oil has detrimental effects on cardiac structure and function in humans is unknown at present. However, claims of beneficial health effects of coconut oil are unsubstantiated. Data of the present study suggest that specific SFA may have direct detrimental effects that are independent of the effects on plasma cholesterol, on body weight, and on insulin sensitivity. Experimental dietary intervention studies with specific medium-chain triglycerides or long-chain triglycerides may unravel the specific culprits of the harmful effects of coconut oil.

## 4. Materials and Methods

### 4.1. In Vivo Experiments Evaluating the Effect of Coconut Oil on the Development of Pressure Overload-Induced Cardiomyopathy

All experimental procedures in animals were performed in accordance with protocols approved by the Institutional Animal Care and Research Advisory Committee of the Catholic University of Leuven (Approval number: P154/2013, 1 October 2013). At the age of 12 weeks, female C57BL/6 mice, originally purchased from Taconic (Ry, Denmark) were fed standard chow (SC) diet (Sniff Spezialdiäten GMBH, Soest, Germany) or SC supplemented with 0.2% (*w*/*w*) cholesterol and 10% (*v*/*w*) coconut oil (CC diet) *ad libitum*. The experimental diet was maintained throughout the entire duration of the experiments. To induce pressure overload, transverse aortic constriction (TAC) was performed at the age of 17 weeks. Briefly, anesthesia was performed with a single intraperitoneal injection of sodium pentobarbital (Nembutal^®^, Ceva Sante Animale, Brussels, Belgium) at a dose of 40–70 mg/kg. Mice were put in supine position and temperature was maintained at 37 °C with a heating pad. A horizontal skin incision of 0.5 cm to 1 cm in length was made at the level of the suprasternal notch. A 2 mm to 3 mm longitudinal cut was performed in the proximal portion of the sternum and the thymus gland was retracted. This allowed visualization of the aortic arch under low-power magnification. A wire with a snare at the end was passed under the aorta between the origin of the right innominate artery and the left common carotid artery. A 7-0 silk suture (Ethicon, Johnson & Johnson, Livingston, Scotland, UK) was snared with the wire and pulled back around the aorta. Subsequently, a bent 27-gauge needle (BD Microlance^®^, BD, Franklin Lakes, NJ, USA) was placed next to the aortic arch and the suture was snugly tied around the needle and the aorta. Afterwards, the needle was quickly removed. The skin was closed and mice were allowed to recover on a warming pad until they were fully awake. The sham procedure was identical except that no constriction on the aorta was applied.

Group assignment at the start of the study was performed at random. At the end of the study, data of all surviving mice were included in the analysis. Endpoint analyses were performed by investigators who were blinded to the group allocation of the animal. Unblinding of animal numbers corresponding to specific allocation groups was performed at completion of measurements.

### 4.2. In Vivo Hemodynamic Measurements

Invasive hemodynamic measurements were performed eight weeks after TAC or after sham operation. Mice were anesthetized by intraperitoneal administration of 1.4 g/kg urethane (Sigma, Steinheim, Germany). Body temperature was maintained with a heating pad and monitored with a rectal probe. An incision in the right carotid artery was made with a 26-gauge needle between a distal and proximal non-occlusive ligation of the artery. A 1.0 French Millar pressure catheter (SPR-67/NR; Millar instruments, Houston, TX, USA) was inserted and advanced to the left ventricle (LV). After stabilisation of the catheter, heart rate, maximal systolic LV pressure, minimal diastolic LV pressure, the peak rate of isovolumetric LV contraction (dP/dt_max_), and the peak rate of isovolumetric LV relaxation (dP/dt_min_) were measured. The end-diastolic LV pressure was calculated manually from the pressure in function of time curves. The time constant of isovolumetric LV pressure fall (tau) was calculated using the method of Weiss et al. [30]. Arterial blood pressure measurements were obtained after withdrawal of the catheter from the LV to the ascending aorta. Data were registered with Powerlab Bridge Amplifier and Chart Software (sampling rate 2000 Hz; ADInstruments Ltd., Oxford, UK).

### 4.3. Blood Sampling

Blood was collected by puncture of the retro-orbital plexus. Anticoagulation was performed with 0.1 volume of 136 mmol/L trisodium citrate and plasma was immediately isolated by centrifugation at 1100× *g* for 10 min and stored at −20 °C.

### 4.4. Plasma Lipoprotein Analysis

Mouse lipoproteins were separated by density gradient ultracentrifugation in a swing-out rotor as described before [31]. Fractions were stored at −20 °C until analysis. Non-HDL cholesterol was determined as the sum of cholesterol within very low-density lipoproteins (VLDL) (0.95 < *d* < 1.006 g/mL), intermediate-density lipoproteins (IDL) (1.006 < *d* < 1.019 g/mL), and low-density lipoproteins (LDL) (1.019 < *d* < 1.05 g/mL) lipoprotein fractions. The cut-off value (*d* = 1.05 g/mL) between LDL and high-density lipoproteins (HDL) for murine samples was chosen based on previous work by Camus et al. [32]. Cholesterol in plasma and lipoprotein fractions was determined with commercially available enzymes (Roche Diagnostics, Basel, Switzerland). Precipath L (Roche Diagnostics) was used as a standard.

### 4.5. Quantification of Insulin and Homeostatic Model Assessment-Insulin Resistance (HOMA-IR)

Murine plasma insulin levels were quantified using the Insulin ELISA kit (Cayman Chemical, Ann Arbor, MI, USA). HOMA-IR values were calculated based on the formula HOMA-IR = (glucose × insulin)/22.5 where glucose is expressed in mmol/L and insulin in mU/L [22,24].

### 4.6. Glucose Tolerance Test

Glucose tolerance test was performed by intraperitoneal injection of glucose (2 g/kg) after 6 h of fasting as described by Hofmann et al. [33] in SC diet mice and in CC diet mice at 13 weeks after the initiation of the diet. Tail blood glucose levels were measured with an Accu-Chek^®^ Active Glucometer (Roche Applied Science, Penzberg, Germany) before (0 min) and at 15, 30, 60, 90, and 120 min after injection.

### 4.7. Analysis of Lipid Peroxidation in Plasma

Measurement of Thiobarbituric Acid Reactive Substances (TBARS) used for quantification of lipid peroxidation was performed according to the instructions of the manufacturer (Cayman Chemical, Ann Arbor, MI, USA).

### 4.8. Quantification of Superoxide Dismutase

Superoxide dismutase activity was analyzed using the Superoxide Dismutase Assay kit (Cayman Chemical, Ann Arbor, MI, USA).

### 4.9. Myocardial Lipid Analysis

Major lipid classes (phospholipids, cholesterol, cholesteryl esters, triglycerides, free fatty acids and sphingomyelin) in the myocardium were analyzed in myocardial lipid extracts with classical (bio)chemical assays [34,35,36,37].

### 4.10. Quantification of Myocardial Protein Levels by Western Blot

Myocardial tissue samples were isolated 56 days after sham operation or TAC and immediately frozen in liquid nitrogen and stored at −80 °C. Tissues were placed in lysing matrix tubes (QBiogene/MP Biomedicals, Solon, OH, USA), mixed with 1 mL of protein extraction buffer containing 10 mmol/L imidazole, 300 mmol/L sucrose, 1 mmol/L dithiotreitol, 1 mmol/L sodium metabisulfite, 25 mmol/L sodium fluoride, 5 mmol/L sodium ethylenediaminetetraacetic acid, 5 mmol/L sodium pyrophosphate, 0.3 mmol/L phenylmethylsulfonyl fluoride, and a protease inhibitor cocktail (Roche Diagnostics Belgium, Vilvoorde, Belgium) [38], and homogenized using the FastPrep24 instrument (MP Biomedicals). Protein concentration was quantified using the Pierce BCA Protein Assay kit (Pierce Biotechnology Inc., Rockford, IL, USA). Equal amounts of proteins were separated on 4–20% Tris-Glycine gradient gels (Bio-Rad Laboratories N.V., Temse, Belgium) and blotted onto polyvinylidene difluoride membranes (Bio-Rad Laboratories N.V.). Membranes were incubated with primary antibodies (Cell Signaling Technologies, Beverly, MA, USA) against Akt, phospho (p)-Akt (Ser/Thr), mitogen-activated protein kinase (MAPK) kinase (MEK) 1/2, p-MEK 1/2 (Ser217/221), p38 MAPK, p-p38 MAPK (Thr180/Tyr182), mammalian or mechanistic target of rapamycin (mTOR), p-mTOR (Ser2481), acetyl-coenzyme A (acetyl-CoA) carboxylase (ACC), p-ACC (Ser79), AMP-activated protein kinase (AMPK)α, p-AMPKα (Thr172), c-Jun N-terminal kinase (JNK), also referred to as stress-activated protein kinase (SAPK)/JNK, p-JNK (Thr183/Tyr185), extracellular signal–regulated kinase (ERK) 1/2, p-ERK 1/2 (Thr202/Tyr204), Smad1, Smad2/3, p-Smad 1/5 (Ser463/465), Smad4, GLUT 4, pyruvate dehydrogenase (PDH), pyruvate dehydrogenase kinase, transforming growth factor (TGF)-β1, and glyceraldehyde 3-phosphate dehydrogenase (GAPDH). Protein expression was detected with Super signal west pico chemilumninescent reagents (Thermo Scientific, Rockford, IL, USA) and quantified using Image lab TM Analyzer software (Bio-Rad laboratories N.V.). All protein levels were normalized to the GAPDH protein level.

### 4.11. Histological and Morphometric Analysis

After hemodynamic analysis, mice were perfused via the abdominal aorta with phosphate-buffered saline (PBS) and hearts were arrested in diastole by CdCl_2_ (100 μL; 0.1 mol/L), followed by perfusion fixation with 1% paraformaldehyde in phosphate buffered saline. After dissection, hearts were post-fixated overnight in 1% paraformaldehyde, embedded in paraffin, and 6 μm thick cross-sections at 130 μm spaced intervals were made extending from the apex to the basal part of the left ventricle. Left ventricle (LV) remodeling was assessed by morphometric analysis on mosaic images of Sirius red-stained heart cross-sections using Axiovision 4.6 software (Zeiss, Zaventem, Belgium). Anterior wall thickness and septal wall thickness were determined. All geometric measurements were computed in a blinded fashion from representative tissue sections of 4 separate regions and the average value was used to represent that animal for statistical purposes [39,40].

To measure collagen content in the interstitium, Sirius Red staining was performed as described by Junqueira et al. [41]. Sirius Red polarization microscopy on a Leica RBE microscope with KS300 software (Zeiss) was used to quantify thick tightly packed mature collagen fibers as orange-red birefringent and loosely packed less cross-linked and immature collagen fibers as yellow-green birefringent. Collagen positive area was normalized to the LV remote area and was expressed as percentage. Any perivascular fibrosis was excluded from this analysis. Perivascular fibrosis was quantified as the ratio of the fibrosis area surrounding the vessel to the total vessel area. Two mid-ventricular sections were studied per animal [42].

Cardiomyocyte hypertrophy was analyzed on paraffin sections stained with rabbit anti-mouse laminin (Sigma; 1/50) by measuring the cardiomyocyte cross-sectional area (μm^2^) of at least 200 randomly selected cardiomyocytes in the LV myocardium. Capillary density in the myocardium was determined on CD31 stained sections using rat anti-mouse CD31 antibodies (BD; 1/500). Relative vascularity in the myocardium was determined as ((capillary density (number/mm^2^)/cardiomyocyte density (number/mm^2^))/cardiomyocyte cross-sectional area (μm^2^)) [6]. Two mid-ventricular cross-sections were analyzed per mouse [39,40].

Immunostaining for 3-nitrotyrosine was performed with rabbit anti-nitrotyrosine antibodies (Merck Millipore, Overijse, Belgium; dilution 1/250).

Apoptosis was quantified on deparaffinized tissue sections using SignalStain^®^ cleaved caspase-3 IHC detection kit (Cell Signaling Technologies, Beverly, MA, USA), which utilizes a polyclonal rabbit antibody to the neoepitope peptide at the end of cleaved caspase-3 [42].

### 4.12. Evaluation of Cardiac Glucose Metabolism by Micro-Positron Emission Tomography (Micro-PET)

Glucose uptake in the myocardium and in the skeletal muscle was quantified by micro-PET using (^18^F)-fluorodeoxyglucose (FDG) as a tracer. Imaging was performed 60 min after tracer administration. Animals were anesthetized by inhalation of 2% isoflurane in 100% oxygen and underwent static imaging for 10 min on a micro-PET Focus 220 scanner (Concorde Microsystems, Knoxville, TN, USA). Images were reconstructed with ordered subset expectation maximization algorithm with 6 iterations (OSEM3D 6i) and analyzed with PMOD v.3.4 (Pmod Technologies, Zurich, Switzerland). To exclude any effect of diurnal variability, micro-PET data acquisition was consistently performed within the same 2 h time frame of the day. The standardized uptake value (SUV) in a specific volume of interest is the ratio between the uptake in this volume versus the average uptake in the whole body. The simultaneous quantification of skeletal SUVs was performed since myocardial glucose metabolism is not necessarily parallel to skeletal and whole-body glucose metabolism [43].

### 4.13. Statistical Analysis

All data are expressed as means ± standard error of the means (SEM). Parameters between four groups were compared by one-way analysis of variance followed by Bonferroni multiple comparisons post-test for comparing sham groups, TAC groups, and sham versus respective TAC groups using GraphPad Instat (GraphPad Software, San Diego, CA, USA). When the assumption of sampling from populations with identical standard deviations was not met, a logarithmic transformation was performed. When the assumption of sampling from populations with Gaussian distributions was not met, a Kruskal–Wallis test was performed followed by Dunn’s multiple comparisons post-test. Parameters between two groups were compared using Student’s *t* test. When indicated, a logarithmic transformation or a non-parametric Mann–Whitney test was performed. The assumption of Gaussian distribution was tested using the Kolmogorov–Smirnov method. Kaplan–Meier survival curves were analyzed by log-rank test using Prism4 (GraphPad Software). A two-sided *p*-value of less than 0.05 was considered statistically significant.

## Figures and Tables

**Table 1:** Total, non-HDL, and HDL cholesterol plasma levels (mmol/L) in C57BL/6 mice fed the SC diet or the CC diet.

Experimental Model	Total Cholesterol	Non-HDL Cholesterol	HDL Cholesterol
C57BL/6 SC diet	1.51 ± 0.11	0.368 ± 0.041	1.14 ± 0.07
C57BL/6 CC diet	1.70 ± 0.10	0.411 ± 0.027	1.29 ± 0.08

SC diet: standard chow diet. CC diet: standard chow diet supplemented with 0.2% cholesterol 10% coconut oil. HDL: high-density lipoproteins. Data are expressed as means ± SEM. (*n* = 10).

**Table 2:** Body weight and organ weights in female C57BL/6 mice eight weeks after sham or TAC procedure in C57BL/6 mice fed the SC diet or the CC diet.

Parameter	SC Diet Sham	CC Diet Sham	SC Diet TAC	CC Diet TAC
Number of mice	16	16	16	16
Body weight (g)	23.2 ± 1.0	24.5 ± 1.1	25.2 ± 0.7	22.6 ± 0.7
Heart weight (mg)	117 ± 7	116 ± 3	236 ± 8 ^†††^	286 ± 9 ^†††,§§§^
Left ventricular weight (mg)	72.6 ± 5.7	74.8 ± 1.8	162 ± 6 ^†††^	178 ± 6 ^†††^
Right ventricular weight (mg)	24.2 ± 1.9	25.3 ± 0.8	39.1 ± 2.1 ^†††^	56.4 ± 2.2 ^†††,§§§^
Lung weight (mg)	147 ± 4	153 ± 4	186 ± 17	328 ± 22 ^†††,§§^
Liver weight (mg)	954 ± 59	974 ± 52	926 ± 25	858 ± 39
Tibia length (mm)	16.9 ± 0.1	17.0 ± 0.2	17.0 ± 0.2	16.8 ± 0.1

SC diet: standard chow diet. CC diet: standard chow diet supplemented with 0.2% cholesterol 10% coconut oil. The CC diet was initiated at the age of 12 weeks. Sham operation or TAC was performed at the age of 17 weeks. Data are expressed as means ± SEM. ^†††^: *p* < 0.001 versus respective sham groups. ^§§^: *p* < 0.01; ^§§§^: *p* < 0.001 versus SC diet TAC.

**Table 3:** Morphometric and histological parameters of the left ventricular myocardium eight weeks after sham operations or after TAC in C57BL/6 mice fed the SC diet or the CC diet.

Parameter	SC Diet Sham	CC Diet Sham	SC Diet TAC	CC Diet TAC
Number of mice	21	13	26	28
Septal wall thickness (μm)	1090 ± 20	1040 ± 20	1520 ± 40 **^†††^**	1590 ± 50 **^†††^**
Anterior wall thickness (μm)	1130 ± 20	1070 ± 20	1660 ± 40 **^†††^**	1680 ± 50 **^†††^**
Cardiomyocyte cross-sectional area (μm^2^)	215 ± 7	199 ± 4	536 ± 27 **^†††^**	595 ± 20 **^†††^**
Cardiomyocyte density (number/mm^2^)	4620 ± 130	4860 ± 150	2760 ± 160 **^†††^**	2440 ± 90 **^†††^**
Capillary density (number/mm^2^)	6380 ± 150	6820 ± 120	5110 ± 220 **^†††^**	4180 ± 190 **^†††,§§§^**
Relative vascularity (μm^−2^)	0.00660 ± 0.00021	0.00713 ± 0.00022	0.00386 ± 0.00017 **^†††^**	0.00296 ± 0.00011 **^†††,§§§^**
Interstitial fibrosis (%)	1.96 ± 0.18	6.59 ± 0.44 ***	10.2 ± 0.9 **^†††^**	19.2 ± 1.3 **^†††,§§§^**
Perivascular fibrosis (ratio)	0.426 ± 0.030	0.573 ± 0.010 *	0.589 ± 0.022 **^†††^**	0.825 ± 0.023 **^†††,§§§^**
Cleaved caspase 3 Positive cells (number/mm^2^)	0.00 ± 0.01	1.25 ± 0.12	23.8 ± 1.1 **^†††^**	38.6 ± 1.6 **^†††,§§§^**

SC diet: standard chow diet. CC diet: standard chow diet supplemented with 0.2% cholesterol 10% coconut oil. The CC diet was initiated at the age of 12 weeks. Sham operations or TAC was performed at the age of 17 weeks. Data are expressed as means ± SEM. *: *p* < 0.05; ***: *p* < 0.001 versus SC diet sham. **^†††^**: *p* < 0.001 versus respective sham groups. **^§§§^**: *p* < 0.001 versus SC diet TAC.

**Table 4:** Hemodynamic parameters in the left ventricle and in the aorta eight weeks after sham operation or after TAC in C57BL/6 mice fed the SC diet or the CC diet.

Parameter	SC Diet Sham	CC Diet Sham	SC Diet TAC	CC Diet TAC
Number of Mice	19	11	18	22
**Left Ventricle**				
Peak systolic pressure (mm Hg)	101 ± 2	97.9 ± 2.2	173 ± 9 **^†††^**	158 ± 6 **^†††^**
End—diastolic pressure (mm Hg)	0.677 ± 0.475	2.65 ± 0.28	2.83 ± 0.79	2.29 ± 0.90
dP/dt max (mm Hg/ms)	12.1 ± 0.4	11.6 ± 0.4	11.9 ± 0.8	9.54 ± 0.45 **^†,§^**
dP/dt min (mm Hg/ms)	−9.91 ± 0.35	−10.1 ± 0.4	−11.5 ± 0.6 **^†^**	−9.71 ± 0.58 **^§^**
Tau (ms)	4.42 ± 0.13	4.99 ± 0.10 *	5.10 ± 0.23 **^†^**	5.34 ± 0.33
Heart rate (bpm)	612 ± 16	631 ± 10	636 ± 13	604 ± 12
**Aorta**				
Mean pressure (mm Hg)	81.6 ± 1.7	78.4 ± 2.1	104 ± 6 **^††^**	97.2 ± 3.4 **^††^**
Systolic pressure (mm Hg)	99.4 ± 1.7	96.8 ± 2.5	172 ± 11 **^†††^**	157 ± 8 **^†††^**
Diastolic pressure (mm Hg)	64.4 ± 2.6	60.7 ± 2.7	61.8 ± 3.7	59.9 ± 3.1

SC diet: standard chow diet. CC diet: standard chow diet supplemented with 0.2% cholesterol 10% coconut oil. The CC diet was initiated at the age of 12 weeks. Sham operations or TAC was performed at the age of 17 weeks. Data are expressed as means ± SEM. *: *p* < 0.05 versus SC diet sham. **^†^**: *p* < 0.05; **^††^**: *p* < 0.01; **^†††^**: *p* < 0.001 versus respective sham groups. **^§^**: *p* < 0.05 versus SC diet TAC.

**Table 5:** Quantification of glucose uptake in the myocardium determined by micro-PET eight weeks after sham operation or after TAC in C57BL/6 mice fed the SC diet or the CC diet.

Parameter	SC Diet Sham	CC Diet Sham	SC Diet TAC	CC Diet TAC
Number of mice	15	16	16	15
Maximal SUV	9.92 ± 0.66	8.86 ± 0.91	15.5 ± 1.1 ^†††^	17.4 ± 0.8 ^†††^
SUV 50%	6.98 ± 0.47	6.17 ± 0.64	10.6 ± 0.8 ^†††^	11.6 ± 0.5 ^†††^
Volume 50% (mm^3^)	99.5 ± 3.9	93.4 ± 2.4	118 ± 9	167 ± 10 ^†††,§§§^
SUV 75%	8.33 ± 0.56	7.56 ± 0.77	13.0 ± 0.9 ^†††^	14.6 ± 0.7 ^†††^
Volume 50% (mm^3^)	31.5 ± 2.4	29.6 ± 2.5	33.3 ± 3.4	37.4 ± 2.4 ^†^
Percent injected dose in myocardium (%)	4.14 ± 0.38	3.37 ± 0.60	8.73 ± 0.99 ^†††^	16.2 ± 0.9 ^†††,§§§^
SUV left quadriceps	0.609 ± 0.047	0.585 ± 0.045	0.551 ± 0.045	0.251 ± 0.027 ^†††,§§§^

SC diet: standard chow diet. CC diet: standard chow diet supplemented with 0.2% cholesterol 10% coconut oil. The CC diet was initiated at the age of 12 weeks. Sham operation or TAC was performed at the age of 17 weeks. Micro-PET analysis was performed at the age of 25 weeks. SUV: standardized uptake value. SUV 50%: average SUV in voxels with a value above 50% of the maximal SUV. SUV 75%: average SUV in voxels with a value above 75% of the maximal SUV. Volume 50%: integrated volume of voxels with a value above 50% of the maximal SUV. Volume 75%: integrated volume of voxels with a value above 75% of the maximal SUV. Data are expressed as means ± SEM. **^†^**: *p* < 0.05; **^†††^**: *p* < 0.001 versus respective sham groups; **^§§§^**: *p* < 0.001 versus SC diet TAC.

**Table 6:** Myocardial lipid levels eight weeks after sham operation or after TAC in C57BL/6 mice fed the SC diet or the CC diet.

Parameter	SC Diet Sham	CC Diet Sham	SC Diet TAC	CC Diet TAC
Number of mice	10	10	10	10
Phospholipids (nmol/mg tissue)	40.6 ± 0.9	40.9 ± 0.7	46.9 ± 1.2 ^†^	40.0 ± 1.6 ^§§^
Cholesterol (pmol/nmol phospholipids)	74.4 ± 2.2	71.6 ± 1.9	65.0 ± 1.4 ^††^	75.0 ± 1.6 ^§§^
Cholesteryl esters (pmol/nmol phospholipids)	2.02 ± 0.20	2.74 ± 0.12 *	1.69 ± 0.13	3.62 ± 0.30 ^§§§^
Triglycerides (pmol/nmol phospholipids)	63.2 ± 5.3	73.2 ± 7.0	62.5 ± 7.2	37.0 ± 2.7 ^†††,§^
Free fatty acids (pmol/nmol phospholipids)	27.5 ± 3.5	36.7 ± 3.3	20.2 ± 4.4	10.4 ± 2.0 ^†††,§^
Sphingomyelin (pmol/nmol phospholipids)	12.3 ± 0.9	11.3 ± 0.6	12.4 ± 0.9	17.1 ± 1.0 ^†††,§§^

SC diet: standard chow diet. CC diet: standard chow diet supplemented with 0.2% cholesterol 10% coconut oil. The CC diet was initiated at the age of 12 weeks. Sham operation or TAC was performed at the age of 17 weeks. Data are expressed as means ± SEM. *: *p* < 0.05 versus SC diet sham. ^†^: *p* < 0.05; ^††^: *p* < 0.01; ^†††^: *p* < 0.001 versus respective sham groups; ^§§^: *p* < 0.01; ^§§§^: *p* < 0.001 versus SC diet TAC.

## Abbreviations

CC diet	0.2% cholesterol 10% coconut oil
FDG	Fluorodeoxyglucose
HDL	High-density lipoproteins
SC diet	Standard chow diet
SFA	Saturated fatty acids
TAC	Transverse aortic constriction
TBARS	Thiobarbituric acid reactive substances
